# *Felis Catus* Gammaherpesvirus 1 DNAemia in Whole Blood from Therapeutically Immunosuppressed or Retrovirus-Infected Cats

**DOI:** 10.3390/vetsci4010016

**Published:** 2017-03-14

**Authors:** Alicia J. McLuckie, Vanessa R. Barrs, Bethany Wilson, Mark E. Westman, Julia A. Beatty

**Affiliations:** 1 Faculty of Science, Sydney School of Veterinary Science, The University of Sydney, Sydney, NSW 2006, Australia; amcl8135@uni.sydney.edu.au (A.J.M.); vanessa.barrs@sydney.edu.au (V.R.B.); mark.westman@sydney.edu.au (M.E.W.); 2 Faculty of Science, School of Life and Environmental Sciences, The University of Sydney, Sydney, NSW 2006, Australia; bethany@cafallstats.com.au

**Keywords:** gammaherpesvirus, retrovirus, co-infection, immunodeficiency, immunosuppression, lymphoma, comparative medicine

## Abstract

Gammaherpesviruses are major co-pathogens of human immunodeficiency virus (HIV) infection, making the interactions between feline immunodeficiency virus (FIV) and *Felis catus* gammaherpesvirus 1 (FcaGHV1) pertinent to both human and veterinary medical research. FIV-infected cats are at increased risk of FcaGHV1 DNAemia and consistently harbor higher FcaGHV1 loads than FIV-uninfected cats. Whether immune deficiencies unrelated to FIV are associated with similar risks is unknown. Using whole blood FcaGHV1 qPCR, we found no difference in the frequency of DNAemia or DNA load in therapeutically immunosuppressed (P1, *n* = 18) or feline leukemia virus (FeLV)-infected (P2, *n* = 57) patients compared with age- and sex-matched controls (C1, *n* = 58; C2, *n* = 57). In contrast, FIV/FeLV-co-infected cats (P3, *n* = 5) were at increased risk of FcaGHV1 DNAemia compared to retrovirus uninfected controls (C3, *n* = 39; *p* = 0.0068), and had a higher median FcaGHV1 DNA load, although the latter was not significant. FIV/FeLV-co-infected cats (P3) had a similar frequency of FcaGHV1 DNAemia reported compared to FIV-infected controls (C4). In conclusion, we found no evidence that cats with therapeutic immunosuppression or FeLV infection were at greater risk of FcaGHV1 DNAemia or had higher FcaGHV1 DNA load in whole blood. The risk of DNAemia in FIV/FeLV-co-infected cats was similar to that documented previously in cats infected with FIV alone.

## 1. Introduction

FcaGHV1 was identified by targeted virus discovery in 2014 [1]. The existence of a gammaherpesvirus in the domestic cat was suggested by observations from the FIV-cat model of HIV infection in humans. Gammaherpesviruses cause persistent infections that are typically clinically latent, unless cell-mediated immunity is compromised, in which case they can cause lymphoproliferative and neoplastic disorders [2]. Both FIV and HIV infections increase the patient’s risk of developing high-grade B cell lymphomas [3,4,5]. Of the HIV-associated lymphomas, 20–100% are associated with the human gammaherpesviruses Epstein–Barr virus (EBV; human herpesvirus 4) and/or Kaposi’s sarcoma associated herpesvirus (KSHV; human herpesvirus 8) [5,6]. Whether FcaGHV1 plays a role in FIV-associated lymphomas is currently under investigation.

FcaGHV1 infection is widely endemic. Circulating virus DNA was detected in 10%–19% of domestic cats from USA, Australia, Europe, and Singapore, and a recent serologic study suggests that the true infection rate is at least double that indicated by molecular studies [7,8,9,10]. To reflect that whole blood viral DNA detection is not equivalent to viremia since it does not distinguish between DNA within cells, virions, or that free in plasma, the term DNAemia is in common usage in the medical literature [11]. Age, sex, neuter status, health status and infectious cofactors have been identified as risk factors for FcaGHV1 DNAemia, with some regional variations [7,8,9]. Older male cats are most likely to be infected and territorial aggression has been suggested as one likely route of FcaGHV1 transmission.

A striking association between FcaGHV1 and FIV infection has been reported in independent studies [7,8]. FIV infection increases the risk of FcaGHV1 detection by five times compared to FIV uninfected controls matched for age and sex [7,8]. In addition, FIV-infected cats harbor FcaGHV1 loads up to 5 times those of FIV uninfected cats [7,8]. The mechanisms and significance of these observations remain to be defined. A similar phenomenon is described in HIV-infected patients. The median EBV load rises early after HIV seroconversion, increasing by five times during the first year, after which stable high EBV loads are maintained for years [12]. The increased EBV load in HIV infection is not correlated with clinical stages, viral load, or CD4 counts, suggesting that the increase in EBV load is not directly related to the degree of immunosuppression [13,14,15].

The effect of therapeutic or other acquired forms of immunosuppression on FcaGHV1 load has not yet been investigated. Cyclosporine, a potent suppressor of cytokine-induced T-cell proliferation, is sometimes used by veterinarians to achieve therapeutic immunosuppression, for example, to treat allergic and immune-mediated disease [16,17]. Feline leukemia virus (FeLV) is an immunosuppressive retrovirus [18]. One study to date has investigated the relationship between FeLV and FcaGHV1; in Singapore, a significant association between FeLV antigenemia and FcaGHV1 detection was identified [7]. A relationship between concurrent FIV and FeLV infections, a combination that results in profound immunosuppression [19], and FcaGHV1 detection has not been reported.

To investigate the effect of immunosuppression on whole blood FcaGHV1 DNA load, we compared the frequency of FcaGHV1 DNAemia and the DNA load by real time quantitative PCR (qPCR) in patients receiving immunosuppressive therapy compared with a FIV and FeLV negative control group matched for age and sex. We next compared FcaGHV1 DNAemia and load in cats with progressive FeLV infection to a similar control group. Lastly, FcaGHV1 DNAemia and load was compared between dual retrovirus infected cats and two control groups: FIV- and FeLV-negative patients and an historical data set of FIV-infected patients, both matched for age and sex.

## 2. Materials and Methods

This study was approved by the University of Sydney Animal Ethics Committee N00/7-2013/3/6029, 2014/626, and N00/1-2013/3/5920. Whole blood in EDTA was obtained prospectively and from an archived biobank from client-owned cats from Australia. Data on age and sex were available for all samples. The following patient groups were recruited:

- Patient Group 1 (P1, *n* = 18) comprised blood samples from cats undergoing therapeutic immunosuppression with cyclosporine at a minimum dose of 2 mg/kg per os. The following inclusion criteria were applied:
  **1.**  treatment with oral cyclosporine (Atopica^®^, Novartis Animal Health Inc., Australia or Neoral^®^, Novartis Pharmaceuticals Pty Ltd., Macquarie Park, NSW, Australia) for ≥7 days AND
  **2.**  clinical remission from the therapeutic indication for cyclosporine AND
  **3.**  negative FIV antibody and FeLV p27 antigen results

Cats were immunosuppressed with cyclosporine alone (*n* = 6) or in combination with prednisolone (*n* = 12) for a minimum of 7 days prior to sampling. All cats were negative for FIV antibody and FeLV p27 antigen on point-of-care (PoC) testing. Median cyclosporine dose was 3.9 mg/kg/d (range 2–10 mg/kg/d) and median prednisolone dose was 1.9 mg/kg/d (range 0.9–3.9 mg/kg/d). Of cats in which blood cyclosporine trough levels had been performed, 7/9 (78%) had levels within (*n* = 5) or exceeding (*n* = 2) the recommended therapeutic range for cyclosporine (250–500 μg/L) [17].

- Patient Group 2 (P2, *n* = 57) comprised blood samples from cats with progressive FeLV infection and negative FIV serology. Progressive FeLV infection was defined as a positive result on a FeLV p27 antigen PoC test and a positive result on an FeLV proviral DNA qPCR [20] on one (*n* = 39) or > one (*n* = 18) occasion.
- Patient Group 3 (P3, *n* = 5) comprised blood samples from cats co-infected with FIV and FeLV. Inclusion criteria were the same as for P2, except that these cats also had a positive FIV serology result using three PoC tests (SNAP^®^ FIV/FeLV Combo test, IDEXX; Anigen Rapid FIV/FeLV Test Kit, Bionote; WITNESS^®^ FeLV-FIV Test Kit, Zoetis) and had never been vaccinated against FIV.

Control Groups 1–3 were selected from a pool of 86 blood samples from cats of known age and sex that tested negative for FIV and FeLV and were not receiving immunosuppressive medication. Control cases were systemically well on physical examination by a veterinarian. Chronic, stable pre-existing conditions, such as periodontal disease Grade I or II (out of V), IRIS Stage I or II (out of IV) chronic kidney disease [21], or medically controlled hyperthyroidism, were permitted to allow age-matching to older cats in the patient groups. The pool was separated into male and female sub-groups to allow sex-matching to patient groups. Samples were selected through random lottery with the operator blinded to FcaGHV1 status. Age-matching was determined by statistical analysis (see below). Control Group 1 (C1, *n* = 58) was matched to P1, C2 (*n* = 57) to P2, and C3 (*n* = 39) to P3. P3 was also compared with C4 (*n* = 21), all of the male cats from an historical data set comprising FIV-infected, FeLV-uninfected cats reported previously [7].

Age and sex data for patient and control groups are summarized in Table 1.

DNA was extracted using a commercial kit (DNeasy^®^ Blood & Tissue Kit, QIAGEN, Australia) and DNA concentration determined using spectrophotometry (Nanodrop^®^ 2000, ThermoFisher Scientific Australia). Conventional PCR for feline gylceraldehye-3-phosphate dehydrogenase (GAPDH) was performed and was positive for all samples [22].

qPCR was used to detect and quantitate FcaGHV1 DNA [1]. Minimum requirements for inclusion of the qPCR result were amplification efficiency between 95%–105% and R^2^ > 0.99. Samples were tested in triplicate and designated positive if all three reactions were positive with a starting quantity of >3 FcaGHV1 DNA copies per reaction. FcaGHV1 DNA load was calculated in viral DNA copies per million host cells [1,23]. qPCR results were analyzed using CFX Manager Software (Bio-Rad Laboratories Inc., Hercules, CA, USA).

Age data for patient groups were compared to respective control groups using either a *t*-test with Welch’s correction or a Mann–Whitney U test, according to group size and normality testing using a Shapiro–Wilk test. *p*-values less than 0.05 were considered significant. Age-matching of patient groups to control groups was confirmed in all cases. The frequency of FcaGHV1 DNAemia was compared between patient and control groups using Fisher’s exact test. FcaGHV1 DNA loads were compared between patient and control groups using a Mann–Whitney U test. Analyses were performed using GraphPad Prism (version 6.04 for Windows, 2014).

## 3. Results

### 3.1. Frequency of FcaGHV1 DNAemia and Relative Risk in Patients Versus Controls

No difference was detected in the frequency of FcaGHV1 DNAemia between P1 (therapeutic immunosuppression) and controls (C1, *p* = 1.000), or between P2 (FeLV infection) and controls (C2, *p* = 0.5572, Table 2). The risk of FcaGHV1 DNAemia was significantly increased in P3 (FIV/FeLV-co-infected) compared to retrovirus negative controls (C3, *p* = 0.0068, relative risk [RR] 5.200), but not compared to FIV-infected controls (C4, *p* = 1.000, Table 2). The prevalence of FcaGHV1 DNAemia was significantly higher in the FIV-infected controls (C4) compared to retrovirus negative controls (C3; *p* < 0.0001, RR = 4.643).

### 3.2. Whole Blood FcaGHV1 DNA Load

**Figure 1:**
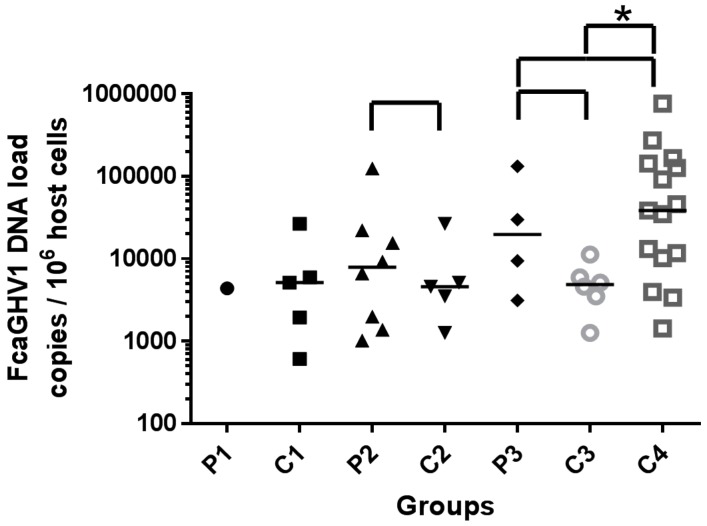
FcaGHV1 viral DNA loads of individuals and median of group (bar). Groups compared statistically shown with 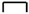 bracket. Significant difference highlighted by *.

Among patients undergoing therapeutic immunosuppression (P1), FcaGHV1 DNA load was similar to that in controls C1 (4392 copies/10^6^ cells versus median 5162, range 611–26,661, IQR 1280–16,309 copies/10^6^ cells, Figure 1). Statistical analyses between P1 and C1 were precluded by the finding of only a single FcaGHV1 positive sample in P1. This cat was being treated concurrently with the recommended dose of cyclosporine (4 mg/kg q24 per os) and with prednisolone (2.3 mg/kg q24 per os). Cyclosporine trough data were not available for this sample.

FcaGHV1 DNA loads of FeLV-infected patients (P2; median 7943, range 1023–124,918, IQR 1524–20,478 copies/10^6^ cells, Figure 1) were not significantly different from those in matched controls (C2; median 4602, range 1261–26,661, IQR 2382–15,912 copies/10^6^ cells, *p* = 0.7242, Figure 1). FcaGHV1 DNA load in patients co-infected with FIV and FeLV (P3; median 19666 copies/10^6^ cells, range 3121–131,840, IQR 4704–106,350, Figure 1) was similar to that in patients infected with FIV only (C4; median 38565 copies/10^6^ cells, range 1427–759,495, IQR 10181–142,676, *p* = 0.4107, Figure 1) and exceeded the load in retrovirus uninfected controls (C3; median 4882, range 1261–11,224, IQR 2942–7273 copies/10^6^ cells), although this relationship did not achieve significance (*p* = 0.2571, Figure 1). When comparing both control groups for P3, the FIV-infected historical group (C4) had a significantly higher median viral load than the retrovirus uninfected matched control group (C3; *p* = 0.0184, Figure 1).

## 4. Discussion

We detected no effect of therapeutic immunosuppression on FcaGHV1 DNAemia or load in whole blood from naturally infected cats. Periodic monitoring of cyclosporine 24-hour whole blood trough levels is clinically indicated because serum cyclosporine concentration is not accurately predicted by dose alone, partly due to low bioavailability of the drug [16]. The requirement for clinical remission as an inclusion criterion suggests that effective immunosuppression was achieved in these cases. The concurrent use of glucocorticoids in two thirds of patients in P1 and inclusion of cats with cyclosporine trough concentrations exceeding the therapeutic range, suggests that a proportion of patients in P1 were profoundly immunosuppressed [24,25]. The prevalence in P1 was the lowest of all groups reported. This may be explained by the high proportion of females (77.8%) in this group. Being male is a significant risk factor for FcaGHV1 DNAemia [7,8] and there was no significant difference in prevalence when P1 was compared to an age- and sex-matched control group (C1). There have been only 25 FcaGHV1 DNAemic female cats identified, in this study and in the literature, out of 417 females tested (6.0%), compared to 170 out of 577 male cats (29.5%) [7,8,9]. We assume the preponderance of females in P1 is due to chance. An increased risk of autoimmune disease in females compared with males is reported in medical studies and some experimental animal models [26,27]. A predisposition to developing autoimmune disease is described in female dogs but is impacted by other variables such as neutering and breed (reviewed [28,29]). No such female bias is reported in cats. In fact, some studies show an overrepresentation of male cats in immune-mediated diseases such as chronic progressive polyarthritis and primary immune-mediated hemolytic anemia [30,31]. An alternate explanation for the low prevalence of FcaGHV1 DNAemia in P1 is that the administration of cyclosporine at doses sufficient to decrease T-cell proliferation may also decrease the circulating FcaGHV1 DNA load below the limit of detection of the qPCR. We have shown previously that FcaGHV1 DNA can be detected in T as well as B lymphocytes [22].

The FcaGHV1 DNA load in the single positive sample in Group P1 was comparable to the median load of FcaGHV1 DNAemic cats in C1. This parallels the situation in human transplant recipients, where therapeutic immunosuppression is accompanied by only minimal changes to EBV load [32]. In these patients, increasing EBV load is an important biomarker for the development of EBV-associated post-transplant lymphoproliferative disease (PTLD). Feline renal transplant recipients treated with cyclosporine face a similar increased risk of malignant neoplasia [25]. The most commonly reported cancer is lymphoma, with feline transplant recipients having 6.7 times higher odds of developing diffuse large B cell lymphoma compared to matched controls [33,34]. Whether a gammaherpesvirus is involved in the etiology of these lymphomas in cats, as it is in over 75% of PTLD in humans, is not yet clear [35].

Our finding that FeLV infection was not associated with increased FcaGHV1 DNAemia in Australia contrasts the significant risk reported previously in Singapore, where 6 of 22 FeLV-infected cats (27%) were FcaGHV1 DNAemic compared to only 10/152 (7%) of controls [7]. Methodological differences, including a tightly matched control population and a larger FeLV-infected group in the current study, are unlikely to fully account for such a disparity. A more plausible explanation is offered by considering that the predominant mode of FeLV transmission may vary between geographic locations. In most regions, epidemiological evidence supports FeLV transmission primarily through non-aggressive or vertical contact, whereas, for FcaGHV1, fighting is proposed as a major route of transmission [7,8,9,36]. In Singapore, if territorial aggression is the principle mode of FeLV transmission, the associated risk of FcaGHV1 DNAemia could reflect co-transmission of these viruses. In support of this theory, the epidemiology of several feline pathogens in Singapore is atypical. For example, the absence of a sex predisposition for either FcaGHV1 or FIV suggests equal participation by males and females in fighting [7]. It has been proposed that behaviors associated with the organized feeding of large populations of street cats in Singapore may play a role in this regional difference. Finally, if FeLV infection per se, rather than co-transmission, was contributing to an increased risk of detectable FcaGHV1 DNAemia, then FeLV infection would likely be accompanied by an increased FcaGHV1 load. However, no association between FeLV infection and FcaGHV1 load was identified either here or in the previous study.

Cats with FIV/FeLV co-infections are difficult to recruit, given the low prevalence of FeLV infection; hence only 5 individuals were available for study. Nonetheless, an association between retrovirus co-infection and FcaGHV1 DNAemia was apparent with triple infections identified in 4 of 5 samples. FIV/FeLV-co-infected patients had the highest FcaGHV1 load of all patient groups, although this was non-significant. The small sample size may have resulted in a type II statistical error, as a significant difference was seen between the FIV-infected historical group (C4) and the retrovirus uninfected matched controls (C3). C4 had a much higher sample number (*n* = 21), improving the power of this analysis, although C4 also had a higher median load than the P3 (FIV/FeLV-co-infected) group.

The associations identified here between FIV/FeLV co-infection and FcaGHV1 are in agreement with those reported previously for FIV infection alone [7,8]. FIV infection carries significant risk of FcaGHV1 DNAemia with median loads 2 to 5 times higher than in retrovirus uninfected controls. This presents a curious parallel to EBV load dynamics in HIV-infected humans. Most adult humans are asymptomatically infected with EBV, which remains latent in some B-cells such that EBV DNA load in whole blood is low or undetectable [37]. However, after HIV seroconversion, EBV loads rise steeply in the first 12 months and high EBV loads are maintained for years [12]. Several reports suggest that this elevation in EBV load is unrelated to the disease stage and CD4 counts [13,14]. Chronic immune activation and loss of T-cell function are proposed to contribute to increased EBV load and, in some patients, to B-cell lymphomagenesis (reviewed in [38]). The immune dysfunction accompanying FIV infection in cats parallels that in HIV-infected humans [39,40]. Further investigation of the interactions between FIV and FcaGHV1 will determine whether the domestic cat provides a much-needed spontaneous model of gammaherpesvirus-associated diseases in HIV patients [41].

In conclusion, we found no evidence that cats undergoing therapeutic immunosuppression or with progressive FeLV infection were at greater risk of FcaGHV1 DNAemia or had higher FcaGHV1 DNA load in whole blood, compared to the controls. FIV/FeLV co-infection was not associated with an increased risk of FcaGHV1 DNAemia greater than that seen in cats infected with FIV alone.

## Figures and Tables

**Table 1:** Summary of patient group data.

Group	(*n* =)	Description	Age (Years)			Sex (*n* =)	
			Median	Range	IQR ^a^	M ^b^	F ^c^
P1	18	therapeutic immunosuppression	5.7	0.7–18.5	3.0–12.3	4	14
C1	58	controls matched to P1	6.5	0.7–20.2	3.7–10.8	13	45
P2	57	FeLV-infected	3.9	0.4–15.3	2.0–8.0	35	22
C2	57	controls matched to P2	4.7	0.5–16.0	2.7–7.9	35	22
P3	5	FIV/FeLV-co-infected	9	0.8–12.5	2.9–11.3	5	0
C3	39	controls matched to P3	6	1.1–16.0	3.4–9.3	39	0
C4	21	historical FIV-infected	12	2.5–17.1	8.5–13.7	21	0

^a^ IQR: interquartile range; ^b^ M: male; ^c^ F: female.

**Table 2:** Prevalence and analyses of FcaGHV1 DNAemia among patient and control groups.

Group	FcaGHV1-Infected	FcaGHV1 Negative	Prevalence (%)	Contingency Table Results			
				Groups Compared	Fisher’s Exact Test ^a^	RR ^b^	95% CI ^c^
P1	1	17	5.6	P1 vs. C1	1.000	0.5556	0.07145 to 4.320
C1	5	53	8.6				
P2	8	49	14.0	P2 vs. C2	0.5572	1.268	0.7892 to 2.039
C2	5	52	8.8				
P3	4	1	80.0	P3 vs. C3	**0.0068**	5.200	2.207 to 12.25
C3	6	33	15.4	C4 vs. C3	**<0.0001**	4.643	2.119 to 10.17
C4	15	6	71.4	P3 vs. C4	1.000	1.120	0.6691 to 1.875

**^a^** *p*-value results; **^b^** RR: relative risk; **^c^** 95% CI: 95% confidence interval of RR.
